# Platelets in Fetal Growth Restriction: Role of Reactive Oxygen Species, Oxygen Metabolism, and Aggregation

**DOI:** 10.3390/cells11040724

**Published:** 2022-02-18

**Authors:** Joanna Nowaczyk, Barbara Poniedziałek, Piotr Rzymski, Dominika Sikora, Mariola Ropacka-Lesiak

**Affiliations:** 1 Department of Perinatology and Gynecology, Poznan University of Medical Sciences, 60-535 Poznan, Poland; mariolaropacka@poczta.onet.pl; 2 Department of Environmental Medicine, Poznan University of Medical Sciences, 61-848 Poznan, Poland; bpon@ump.edu.pl (B.P.); dominika.sikora97@o2.pl (D.S.); 3 Integrated Science Association (ISA), Universal Scientific Education and Research Network (USERN), 60-806 Poznań, Poland

**Keywords:** platelets, oxidative stress, pregnancy, oxygen consumption, reactive oxygen species

## Abstract

Fetal growth restriction (FGR) is mainly caused by failure of the uteroplacental unit. The exact pathogenesis remains unclear. The cause is thought to be related to abnormal platelet activation, which may result in microthrombus formation in the small vessels of the placenta. Reactive oxygen species (ROS) may initiate the pathological process of platelet activation. This study aimed to evaluate selected platelet parameters in pregnancy complicated by FGR and relate them to the severity of hemodynamic abnormalities. A total of 135 women (pregnant with FGR, with an uncomplicated pregnancy, and non-pregnant) were enrolled to study different platelet parameters: count (PLT), mean volume (MPV), ROS levels, intracellular oxygen level, oxygen consumption, and aggregation indices. No abnormalities in PLT and MPV were found in the FGR group, although it revealed increased ROS levels in platelets, lower platelet oxygen consumption, and intraplatelet deprivation. Aggregation parameters were similar as in uncomplicated pregnancy. No significant relationships were observed between hemodynamic abnormalities and the studied parameters. Platelets in pregnancies complicated by FGR may reveal an impaired oxidative metabolism, which may, in turn, lead to oxidative stress and, consequently, to an impaired platelet function. This study adds to the understanding of the role of platelets in the etiology of FGR.

## 1. Introduction

Fetal growth restriction (FGR) is a common pregnancy complication, affecting approximately 4–6% of all pregnancies, and has been associated with various adverse perinatal outcomes [1]. FGR has been defined as the inability of the fetus to reach its intrinsic (i.e., genetically determined) growth potential [2]. It has been classically identified at an estimated fetus weight (EFW) below the 10th percentile for gestational age [3]. Figueras and Gratacos proposed newer criteria according to which FGR should be diagnosed based on an EFW less than the 10th percentile accompanied by the presence of any Doppler blood flow abnormalities associated with a poorer perinatal outcome including the Doppler cerebroplacental ratio (CPR), uterine artery Doppler (UtA PI), or an EFW less than the 3rd percentile [2]. The leading cause of FGR is a failure of the uteroplacental unit, which results in decreased blood flow across the placenta [4,5]. This condition likely results from impaired normal trophoblast invasion of the uterine vasculature early in pregnancy [6,7]. The normal conversion of the spiral arteries into low-resistance vessels is impaired, resulting in reduced delivery of oxygen and nutrients to the fetus [8]. 

The exact pathogenesis of this process remains unclear. Still, given the histopathological changes found in the placenta [9], the cause is related to abnormal platelet activation [10], which may result in excessive platelet aggregation and microthrombus formation in the small vessels of the placenta, limiting the area of maternal–fetal exchange. It appears that reactive oxygen species (ROS), a consequence of oxidative stress, may initiate the pathological process of platelet activation, playing an essential role in the etiopathogenesis of this condition [11]. Knowledge of placental abnormalities in pregnancies complicated by FGR remains insufficient. This results in a lack of effective prevention and treatment of this condition and is often associated with the occurrence of preterm labor with its consequences. On the other hand, an unfavorable intrauterine environment through chronic fetal hypoxia affects the future health status and quality of life. This is due to the altered gene expression and reprogramming of metabolic processes [12], which contribute to cardiovascular disease, hypertension, and polymetabolic syndrome in adulthood [1]. FGR is also one of the leading causes of intrauterine fetal death (IUFD) late in pregnancy [2,13]. 

Given the importance of platelets in regulating hemostasis and thrombosis [14] and the fact that procoagulant platelet activity may lead to impaired placental circulation implicated in the development of FGR [15], it is necessary to study various biochemical aspects of thrombocyte function. 

The present study aimed to analyze the ROS levels, oxygen consumption, and aggregation in platelets in healthy pregnancies and those complicated by FGR and to compare these parameters with non-pregnant healthy women of reproductive age. We hypothesized that the group with FGR would reveal increased intraplatelet ROS content, impaired oxygen metabolism, and aggregation abnormalities. 

## 2. Materials and Methods

### 2.1. Study Participants

The research project was divided into two stages. In the first stage, the ROS levels, oxygen consumption, and intracellular oxygen deficiency were studied in platelets from pregnant women. Platelet aggregation was analyzed in another subset of pregnant women in the second stage. All studied women were patients at the Department of Perinatology and Gynecology of Poznan University of Medical Sciences.

Blood samples for platelet isolation and analyses were obtained from pregnant Caucasian females hospitalized at the Gynecologic and Obstetrical University Hospital in Poznan, Poland. In addition, a subset of non-pregnant females was also recruited to collect samples for comparative analyses.

The present research enrolled 135 Caucasian women divided into 3 groups: (1) patients with pregnancy complicated by FGR (FGR group; *n* = 44); (2) patients with uncomplicated pregnancy (control pregnancy; *n* = 67); (3) non-pregnant healthy women of reproductive age (non-pregnant control; *n* = 24). For the comparisons of the three groups, the minimal total sample size was calculated at the level of *n* = 66, assuming a strong effect (0.4), error rate α = 0.05, and power of a test (1-β) = 0.8. For group comparisons, the minimal total sample size was calculated at the level of *n* = 58, assuming a strong effect (0.8), error rate α = 0.05, power of a test (1-β) = 0.8, and group inequality (*n*2/*n*1) = 2. These assumptions were met by the sample employed in our study.

The inclusion criterion for group 1 was pregnancy complicated by fetal growth restriction (gestational age ≥ 23 weeks). The FGR was defined according to a Delphi procedure and international consensus [16]. The diagnosis of FGR and clinical severity were determined according to the protocol proposed by Figueras and Gtaracos [2], using a calculator created for this purpose and posted online at https://medicinafetalbarcelona.org/calc/ (accessed 7 February 2022). The exclusion criteria for group 1 included: fetal congenital anomalies, fetal chromosomal aberrations and other genetic abnormalities, presence of TORCH infection, multiple pregnancies, coexistence of chronic maternal diseases except for arterial hypertension, and lack of confirmed infections. The second group was recruited to match group 1 with age and BMI. This was conducted to exclude the evidenced effect of age and increased BMI on FGR risk [17]. Group 2 included women in uncomplicated pregnancy with normal fetal growth (gestational age ≥ 23 weeks), no fetal anomalies, and a lack of maternal chronic diseases. Group 3 was recruited to match both pregnancy groups by age and BMI. The inclusion criteria for group 3 included: age < 35 years old, non-smoking, normal BMI, and normal hematological parameters. The research protocol was approved by the Local Bioethical Committee of the Poznan University of Medical Sciences, Poznan, Poland (approval number: 524/2017; issue date 11 May 2017) and was performed in accordance with the ethical standards as described in the 1964 Declaration of Helsinki and its later amendment. Written informed consent was obtained from each patient. 

### 2.2. Platelet Count and Volume

Venous blood was collected in tubes containing ethylenediaminetetraacetic acid as an anticoagulant. The automated blood cell analyzer, Sysmex-XN1000 (Mississauga, ON, Canada), was employed to measure the platelet count. Mean platelet volume (MPV) was calculated according to the formula:

$$\mathrm{MPV}\;\left(\mathrm{fL}\right)=\frac{\mathrm{plateletcrit}\;\left[\%\right]}{\mathrm{platelet}\;\mathrm{count}\;\left[\times 10^{9}\;\mathrm{per}\;L\right]}\times 10^{5}$$

in which plateletcrit was defined as a ratio of the platelet volume to the whole blood volume.

### 2.3. Assessment of Oxidative Metabolism and Oxidative Stress

The general principle of most methods for detecting and monitoring intracellular ROS levels is to convert a nonfluorescent compound, which is permeable through the cell membrane, to a compound that gives fluorescence under the action of ROS inside the cell. The fluorescence signal is thus proportional to the intracellular ROS level. It can be detected using a multi-plate reader equipped with a detector for excitation and emission of a signal of a specific wavelength. Of importance is the fact that ROS have low stability; thus, analyses should only be performed on fresh cells and as soon as possible after cell collection [18,19,20].

#### 2.3.1. Platelet Isolation

From each patient, blood was drawn from a peripheral vein using minimal stent compression for testing. Blood was collected directly into closed system tubes containing the active anticoagulant sodium citrate with citric acid and dextrose (ACD) (BD Vacutainer^®^ tubes with ACD, Erembodegem, Belgium). The final blood anticoagulant concentrations were sodium citrate (1.89 mg/mL), glucose (2.1 mg/mL), citric acid (0.69 mg/mL), and potassium sorbate (0.03 mg/mL). Large gauge needles, 21 G or larger, were used to reduce the risk of potential platelet activation during sampling. Care was taken to ensure that the tube was filled with blood to the manufacturer’s labeled level. The blood was then mixed with the anticoagulant by gently inverting the tube 4–5 times, without shaking. Immediately after blood collection, the tubes with the test material were transported at room temperature in an isothermal container, in an upright position, to the laboratory. Immediately after the delivery of the material, further analyses were performed no later than 40 min from the blood collection. Immediately after transferring the material to the laboratory, platelet-rich plasma was prepared by centrifugation, from which platelets were isolated according to the method described in our previous study [21].

#### 2.3.2. Assessment of Intracellular Reactive Oxygen Species in Platelets

A method based on dichlorofluorescein 2′,7′-dichlorofluorescein diacetate (DCFDA), available with the Cellular ROS Assay Kit^®^ (Abcam, Cambridge, UK), was used to examine the levels of reactive oxygen species in the platelets of female patients [18]. DCFDA is a fluorogenic dye that penetrates the cell membrane and enables the measurement of cellular level of various ROS. The compound is digested to a reduced form by cellular esterases after prefiltration through the cell membrane. This molecule is then oxidized by a series of reactive oxygen species to 2′,7′-dichlorofluorescein (DCF). DCF is a highly fluorescent compound that can be detected by fluorescence spectroscopy at maximum excitation and emission spectra of 485 and 535 nm, respectively. 

The isolated platelets (1 × 10^6^ cells/mL) were placed in a buffer and centrifuged and then incubated with 20 µM DCFDA solution at 37 °C for 30 min in the dark. Platelets were then centrifuged again, and the supernatant was removed to eliminate excess DCFDA. Cells were placed in a 96-well plate (1.5 × 10^5^ cells/50 µL/well). Immediately after seeding the cells onto the plate, the fluorescence level was measured using a multidetector reader equipped with fluorescence detectors with a readout signal of Ex/Em: 485/535 nm. Results were expressed in fluorescence units (FUs). DCFDA is not compatible with fixed samples; thus, stained cells must be measured alive.

#### 2.3.3. Assessment of Oxygen Consumption in Platelets

The assessment of oxygen consumption in isolated platelets was performed with the MitoXpress^®^ Xtra assay (Agilent, Santa Clara, CA, USA). It enables cellular respiration assessment by measuring cells’ extracellular oxygen consumption rates (OCRs) in real time. This method allows for determining the rate of cellular respiration for metabolic characterization. The reagent used in the assay is an oxygen-sensitive fluorophore based on biopolymers. It is chemically stable and inert, soluble in water, and impermeable to cells. The fluorescence signal released by the probe is blanked out by oxygen, so the lower the concentration of molecular oxygen around the cell, the higher the fluorescence signal recorded by a multiplate reader equipped with appropriate excitation/emission filters. The higher the fluorescence signal of the probe, the more oxygen the cell consumes and uses, reflecting the activity of the mitochondrial respiratory chain. Suspended in solution, the isolated platelets were placed in a 96-well microplate at a 5–6 × 10^5^ cells/well density. After adding 10 μL of MitoXpress^®^ Xtra solution to each well, each well was immediately sealed by gently applying 100 μL of HS mineral oil, preheated to a measurement temperature of 37 °C. Fluorescence levels were then measured using a multidetector reader equipped with fluorescence detectors with a readout signal of Ex/Em: 380/650. The results are expressed as FUs.

#### 2.3.4. Assessment of Intraplatelet Oxygen Level

The MitoXpress^®^ Intra assay (Agilent, Santa Clara, CA, USA) was used to quantify the intracellular oxygen concentration in platelets. This measurement allows for determining the state of intracellular oxygenation or hypoxia. The assay method employs a patented oxygen-sensitive nanoparticle probe that penetrates the cell. The fluorophore used in the test is chemically stable and inert. Oxygen suppresses the phosphorescent emission of the probe; thus, the measurement signal (Ex/Em: 380/650 nm) is inversely proportional to the intracellular oxygen concentration. The probe emission was monitored using fluorescent plate readers. The isolated platelets were placed in a 96-well microplate so that the cell density was typically 30,000–80,000 per well. Two wells were left free of MitoXpress^®^ Intra reagent addition as blank controls. One hundred microliters of properly prepared MitoXpress^®^ Intra solution were added to each well. The instrument was then heated to 37 °C. Fluorescence levels were immediately measured using a multidetector reader equipped with fluorescence detectors. The fluorescence baseline signal was measured. The results were expressed as FUs.

### 2.4. Impedance Aggregometry (Multiplate^®^)

The platelet aggregation was assessed with the Multiplate^®^ Platelet Function Testing 5.0 Analyzer (Roche, Rotkreuz, Switzerland), a multi-channel platelet function analyzer based on the impedance aggregation method. Multiplate^®^ assesses platelet function in whole blood within 10 min of starting the analysis. Whole blood was collected into hirudin tubes, then diluted with saline and, after the addition of tests containing platelet activators or inhibitors, analyzed. During the aggregation process, platelets accumulated on electrodes, resulting in a change in electrical resistance between them. The system detects the change in electrical conductivity at two independent electrodes, and the change in resistance during analysis was continuously recorded. Multiplate^®^ uses an innovative “multiple electrode aggregation”, which means a double determination is performed during each measurement. The results were expressed as the area under the curve (AUC) using average values from two independent measurements (two electrodes). The AUC parameter depends on both the total impedance increase and the kinetics of the aggregation process, and it is the parameter that best characterizes the aggregation process during the Multiplate^®^ test. For Multiplate^®^ testing, blood was drawn from a peripheral vein from each patient using minimal stent compression. Blood was drawn directly into tubes with a closed system containing hirudin at a concentration > 15 µg/mL (S-Monovette^®^ Hirudin 1.6 mL, Sarstedt, Nümbrecht, Germany). Large gauge needles, 21 G or larger, were used to reduce the risk of potential platelet activation during sample collection. Care was taken to ensure that the tube was filled with blood to the manufacturer’s indicated level. The blood was then mixed with the anticoagulant by gently inverting the tube 4–5 times, without shaking. Tests were performed between 30 and 180 min after blood collection. Using an automatic pipette, 300 µL of whole blood mixed with anticoagulant (hirudin) was measured and placed in a Multiplate^®^ Test Cells containing two electrodes and a magnetic stirrer. After diluting the blood with the same volume of 0.9% NaCl solution, the whole was incubated for 3 min at 37 °C. Then, 20 µL of the solution containing a platelet agonist was added using ASPItest or TRAPtest, and electrode impedance changes were recorded continuously by the apparatus for 6 min. A blood sample from each patient enrolled in the study was subjected to two ASPItest and TRAPtest analyses, and the results of both measurements were expressed in AUC units.

### 2.5. Calculations and Statistical Analyses

The statistical analyses were performed with the IBM SPSS Statistics 23 package (IBM, New York, NY, USA). The normality of the distribution was evaluated with the Kolmogorov–Smirnov test. For variables differing from Gaussian distribution, additional verification of the level of skewness was performed. Based on this, parametric or non-parametric statistical methods were employed: Student’s *t*-test for independent samples, Mann–Whitney U test for asymmetric variables, Pearson’s *r* coefficient correlation analysis, Spearman’s ρ rank correlation analysis, one-way analysis of variance in a between-groups scheme, Kruskal–Wallis test, χ^2^ test, and Fisher’s exact tests. A *p* < 0.05 was considered statistically significant; probability scores at the 0.05 < *p* < 0.1 level were interpreted as significant at the level of the statistical trend.

## 3. Results

### 3.1. Demographic Characteristics

Demographic characteristics of the studied group, types of delivery, and general platelet parameters are given in Table 1. In the results of the platelet count and MPV between the studied groups, no results were reported, even at the level of the statistical trend. The mean ± SD birth weight in FGR and uncomplicated pregnancy groups was 1966.3 ± 614.9 and 3449.9 ± 468.9 g, respectively. Within the FGR group, 79.1% of fetuses were classified as stage I, 7.0% as stage II, 11.6% as stage III, and 2.3% as stage IV.

### 3.2. Assessment of Aerobic Metabolism and Oxidative Stress in Platelets 

#### ROS Levels, Oxygen Consumption, and Intracellular Oxygen Deficit in Platelets 

Table 2 shows the results of the analyses of ROS levels, oxygen consumption measurements, and intracellular oxygen deficit levels in the studied groups. The highest ROS levels were seen in the FGR group, and in the uncomplicated pregnancy, ROS levels were lower than in the FGR group but higher than in the non-pregnant group. The FGR group had lower levels of platelet oxygen consumption than the other two groups (*p* < 0.001). In turn, these did not differ even at the level of statistical trend, *p* = 0.840. The intracellular oxygen deficit level in the FGR group was higher than in the non-pregnant group (*p* = 0.002) and in the uncomplicated pregnancy group (*p* = 0.014). These, in turn, did not differ even at the level of statistical trend, *p* = 0.718.

### 3.3. Impendence Aggregometry

The results of the impendence aggregometry tests are shown in Table 3. There were no differences between the studied groups even at the level of statistical trend (Table 3).

### 3.4. Correlation between FGR Grade and ROS Levels, Oxygen Consumption, Intracellular Oxygen Deficit, and Aggrometric Parameters

Correlations between the degree of FGR and the level of ROS, oxygen consumption, intracellular oxygen deficit, and the results of ASPI and TRAP tests were examined. In all cases, the studied parameters did not reveal statistically significant relationships with FGR grade (*p* > 0.05 in all cases).

## 4. Discussion

The present study’s results demonstrate that maternal platelets in pregnancies complicated by FGR reveal increased ROS levels and impaired oxygen metabolism. Although no differences were found in platelet count and MPV, these findings indicate that impaired platelet function plays a role in FGR. Examination of intraplatelet ROS and oxygen consumption during the clinical routine is currently challenging but restoring the redox homeostasis can be considered a potential target of pharmacological interventions to prevent FGR. This is particularly important because FGR is one of the leading causes of perinatal and neonatal morbidity and mortality and one of the most common causes of adverse long-term outcomes. At the same time, its clinical management still remains a very challenging task despite advances in obstetric and neonatal care. An adverse intrauterine environment, through its impact on prenatal programming, predisposes the newborn with FGR to long-term health problems such as impaired neurological development and an increased risk of cardiovascular disease [22] and metabolic syndrome [23] that extend throughout life [24]. 

The precise pathophysiological pathways underlying FGR are not fully understood [25]. Some observations suggest the role of oxidative stress [26] and a local thrombogenic state characterized by the formation of microthrombi in placental vessels, which may result from excessive activation and aggregation of platelets [27]. It should be emphasized that both too high of an oxygen concentration and the presence of hypoxia during the process of trophoblast invasion can induce the occurrence of oxidative stress, understood as an imbalance between antioxidants and reactive oxygen species, such as superoxide anion (O_2_^●−^) and hydrogen peroxide (H_2_O_2_) [25]. Platelet redox imbalance may, in turn, not only lead to oxidative damage of thrombocytes. Growing evidence shows that ROS play a profound role in the regulation of platelet activation [18,28,29]. Different types of ROS can support collagen-, thrombin-, ADP-, and arachidonic acid-dependent activation of platelets, trigger tyrosine phosphorylation of β3, or scavenge platelet- and endothelium-derived nitric oxide, subsequently limiting its antiplatelet effect [30,31,32,33,34,35]. Therefore, the assessment of ROS production and cellular metabolism may be necessary for understanding the role of platelets in pregnancies complicated by FGR. In our study, the decidedly highest ROS levels were found in platelets from women with pregnancies complicated by FGR.

Except for ROS levels, we also examined intraplatelet oxygen metabolism. Oxygen is a crucial substrate and energy source for the cell, regulating its metabolism and gene expression. Molecular oxygen is the ultimate acceptor of the electron transport chain and is therefore a highly informative marker of cellular metabolism and mitochondrial function. Mitochondrial function is closely related to biological oxidation processes. Therefore, these cellular organelles play an essential role in generating intracellular ROS, being responsible for 90% of their endogenous production [36,37]. A cell’s oxygen consumption measurement is probably the most informative parameter for assessing its metabolism, because it allows a direct and specific evaluation of the functioning of the electron transport chain underlying oxidative phosphorylation and cellular metabolism [38,39,40]. Traditionally, oxygen consumption in isolated mitochondria and whole cells was performed using a Clark electrode [40]. Currently, convenient and rapid methods using fluorescent probes to measure extracellular oxygen concentration are used for this purpose, whereby average values can be monitored in areas immediately surrounding the cell after sealing the cell suspension with mineral oil [41,42]. In our study, a lower thrombocyte respiratory activity was expressed by reduced platelet oxygen consumption from women with pregnancies complicated by FGR compared to a group of uncomplicated pregnancies and healthy non-pregnant women, both of which did not differ from each other. Our findings indicate the potential abnormalities in the mitochondrial respiratory chain leading to impaired platelet function in pregnancy complicated by FGR. 

Another test that can provide valuable information about the bioenergetic status of cells, including platelets, is the measurement of intracellular oxygen concentration. Advances in developing cell-penetrating nanoparticle probes for O_2_ detection, such as MitoXpress Intra^®^, which stains a variety of mammalian cell types, have enabled the analysis of cellular oxygenation levels in situ [43,44]. The MitoXpress Intra^®^ probe is taken up by the cell by endocytosis, and the luminance level of the probe measured on the plate reader correlates with the level of intracellular O_2_ concentration (inverse relationship) [44,45]. The transition from a well-oxygenated cell to anoxia causes a significant increase in phosphorescence. In mammalian cells, oxygen concentrations at appropriate levels must be maintained to achieve cellular homeostasis. Even a moderate reduction in the intracellular oxygen concentration makes it difficult for cells to fulfill their physiological function [46,47]. The present study found that platelets from women with pregnancies complicated by FGR had lower intracellular oxygen levels than platelets from uncomplicated pregnancies and non-pregnant women, which may indicate that platelet hypoxia in pregnancies complicated by FGR is the cause of abnormal thrombocyte function. The oxygenation level of platelets from women with uncomplicated pregnancies was not significantly different from that of non-pregnant women. Further studies are necessary to understand the factors inducing the hypoxic state in maternal platelets. Considering that studies show that mitochondria may not only play an energetic role in platelet activation but contribute to this process through ROS generation, mitochondrial permeability transition, and collapse of mitochondrial membrane potential [48], targeting platelet mitochondria may potentially prevent abnormalities in oxygen metabolism and associated impaired platelet function.

In addition to ROS and oxygen metabolism, we also examined whether platelet count and MPV are altered in pregnancy complicated by FGR. It is known that in healthy pregnant women, some platelet parameters undergo physiological changes. Such changes include a decrease in PLT with the duration of pregnancy due to the hemodilution and increased activation and destruction of platelets during passage through the placenta, beginning in the second trimester of pregnancy. Pregnancy is a state of increased platelet turnover resulting from increased platelet activation, consumption, and replacement. Consequently, accelerated platelet production to maintain proper hemostasis results in increased platelet volume MPV [49]. Mine Kanat-Pektas et al. conducted a study to determine whether mean platelet volume determined in the late first trimester of pregnancy can be used to predict the occurrence of preeclampsia and FGR. They showed that MPV values of 10.5 fL or more predict FGR with 82% sensitivity and 60% specificity. Thus, increased MPV in the late first trimester of pregnancy reflects increased platelet activation, which may be responsible for impaired uteroplacental circulation [50]. In our research, both MPV and PLT parameters were analyzed. These parameters did not differ significantly between women with pregnancy complicated by FGR and the control group of women with uncomplicated pregnancies. It is essential to highlight that our research was performed in the late second and third trimester of pregnancy, compared to the study by Mine Kanat-Pektas et al., completed in the late first trimester of pregnancy. The differences between the results obtained in this study and the data in the literature can also be explained by the methods used to measure MPV. The different techniques can result in differences in measurements of up to 40% [50]. Altered platelet function may be involved in the development of placental insufficiency, given the results achieved during antiplatelet therapy [51,52]. However, published data on the association between specific platelet parameters and adverse obstetric outcomes are still limited. Excessive platelet activation early in pregnancy may promote altered endothelial cell phenotype, increased leukocyte recruitment, and inflammation at the maternal–fetal interface, which may lead to the development of pregnancy complications of placental etiology such as preeclampsia and FGR [27].

Multiplate^®^ Assay, employed in the present study, has not been used regularly in clinical practice regarding pregnant women. Blomqvist et al. used it to evaluate platelet aggregation in healthy women with normal pregnancies, resulting in a healthy baby. Aggregation was also compared in pregnant and postpartum women compared to healthy non-pregnant women of childbearing age, where a reduction in aggregation was demonstrated under the influence of arachidonic acid (ASPI test) and thrombin receptor activating peptide (TRAP test) in the pregnant and postpartum groups [53]. In addition, Mustafa Can et al. used Multiplate^®^ to compare collagen-mediated platelet aggregation in normal pregnancy with pregnancy complicated by preeclampsia, demonstrating that this assay fails to detect differences between the study groups [54]. Another study using Multiplate^®^ was conducted by Navaratnam et al. to evaluate the occurrence of platelet resistance to acetylsalicylic acid (ASA) used prophylactically in women with pregnancies at high risk for preeclampsia. No reduced sensitivity to acetylsalicylic acid in pregnant women at high risk for preeclampsia was found [53,55]. In our study using impedance aggregometry, we demonstrated that there was no statistically significant difference in the degree of platelet aggregation assessed in whole blood obtained from pregnant women in response to arachidonic acid (ASPI test) and thrombin receptor activating peptide (TRAP test) between patients with pregnancies complicated by FGR and those with uncomplicated pregnancies. There was a statistically significant association between the week of gestation at the study and the degree of platelet aggregation in response to arachidonic acid (ASPI test) in women with pregnancies complicated by FGR. In this group, arachidonic acid-dependent platelet aggregation decreased with the advancement of pregnancy, which is consistent with findings in preeclampsia and may support the hypothesis of increased platelet activation early in pregnancy in this complication [49,56]. To our knowledge, the only study evaluating platelet aggregation in pregnancies complicated by FGR was a study comparing platelet aggregation in pregnancies complicated by FGR with associated hypertension with pregnancies complicated by FGR without associated hypertension using the Ultra-Flo 100 platelet counter. In the hypertensive FGR group, the collagen and ADP-dependent aggregation levels before delivery were almost 50% lower than in normal pregnancy. In contrast to the hypertensive FGR group, the normotensive FGR group showed similar ADP- and collagen-dependent aggregation levels to those found at 36 weeks of normal pregnancy [10]. In our study, there was no correlation between the degree of platelet aggregation in response to arachidonic acid (ASPI test) and thrombin receptor-activating peptide (TRAP test), MPV, and clinical stage of FGR.

Study limitations should be stressed. Firstly, this was experimental research in which platelet parameters were examined ex vivo, while in humans, the function of thrombocytes remained in strict interactions with various cellular and chemical blood constituents [57]. Secondly, the sample size was relatively small, as it integrated clinical routine with experimental laboratory research. However, this size was sufficient to reveal significant findings. Nevertheless, the results encourage further studies on a larger sample size. Third, the results demonstrated a relationship between increased intraplatelet ROS levels and abnormal oxygen consumption and pregnancy complicated by FGR, but they do not necessarily imply causation. Further studies are necessary to understand whether oxidative stress and impaired oxygen metabolism in maternal platelets are a cause or consequence of FGR. Fourth, the evaluation of studied parameters was performed at one time point. In the future, longitudinal studies are necessary to understand the critical points at which platelets’ ROS levels and oxygen consumption can be affected the most during a pregnancy complicated by FGR. Last but not least, platelet aggregation was based on two agonists, while there were other platelet activation pathways (e.g., ADP- and collagen-dependent [58]), the meaning of which should be subject to studies in the future. 

## 5. Conclusions

This study examined several platelet-related parameters in pregnancy complicated by FGR. Basic parameters (i.e., PLT and MPV) did not reveal any abnormalities in the studied group. However, platelet ROS levels were significantly increased in FGR, indicating oxidative stress, which promotes the activation and aggregation of thrombocytes thus initiating the inflammation. Increased levels of ROS in pregnancies complicated by FGR may also be associated with impaired vasodilation and, thus, impaired blood flow and the potential development of hypoxia. The lowest thrombocyte oxygen consumption was observed in pregnancies complicated by FGR, suggesting impaired platelet oxygen metabolism in this group. The group of patients with pregnancies complicated by FGR had the highest deficit in intracellular thrombocyte oxygen levels, which may be related to impaired oxygenation and/or high levels of oxidative stress in these pregnancies. In a pregnancy complicated by FGR, no significant platelet aggregation abnormalities were found using the aggregation assay methods described above. In pregnancies complicated by FGR, there was no correlation between abnormal platelet oxygen metabolism in pregnant women and fetal hemodynamic abnormalities. The study adds to the general understanding of the role of platelets in pregnancy complicated with FGR and warrants further research in this field.

## Figures and Tables

**Table 1:** Demographic characteristics of the studied group of pregnant women (*n* = 111), types of delivery, and general platelet parameters.

	FGR (*n* = 44)	Uncomplicated Pregnancy (*n* = 67)	*p*-Value
**Age** (years), mean ± SD	28.6 ± 5.1	30.39 ± 5.01	0.075
**Number of pregnancies**, mean ± SD	1.5 ± 0.85	2.1 ± 1.2	**0.001**
**BMI** (kg/m^2^), mean ± SD	27.7 ± 5.7	26.7 ± 3.3	0.298
**Delivery** (week of pregnancy), mean ± SD	35.8 ± 3.7	39.2 ± 1.0	**<0.001**
**Preterm birth** (%)	34.9	3.1	<0.001
**Spontaneous labor** (%)	30.2	52.3	
**Cesarean section** (%)	67.4	35.4	0.003
**Vacuum extractor/forceps surgery** (%)	2.3	12.3	
**Cesarean section for urgent indications** (%)	44.0	4.3	0.002
**Platelet count** (G/L), mean ± SD	220.6 ± 50.3	212.6 ± 50.6	0.422
**MPV** (fL), mean ± SD	11.3 ± 0.9	10.9 ± 1.2	0.086

**Table 2:** ROS levels, oxygen consumption, and intracellular oxygen deficit in the platelets isolated from different groups of women.

	Non-Pregnant Control (*n* = 24)		FGR (*n* = 44)		Uncomplicated Pregnancy (*n* = 67)		
	Mean	SD	Mean	SD	Mean	SD	*p*-Value
ROS (FU)	173.1	60.1	292.1	32.8	240.3	33.1	<0.001
Oxygen consumption (FU)	35,818.3	2730.8	26,191.8	4925.7	36,722.9	4525.5	<0.001
Intracellular oxygen deficit (FU)	949.2	119.8	1131.0	248.2	980.5	104.1	**0.002**

**Table 3:** Results of ASPI and TRAP tests in the studied groups.

	FGR (*n* = 44)		Uncomplicated Pregnancy (*n* = 67)		
	Mean	SD	Mean	SD	*p*-Value
ASPI test (AU× min)	252.62	161.37	217.60	117.25	0.330
TRAP test (AU× min)	434.79	212.62	403.23	162.87	0.534

## Data Availability

The data presented in this study are available from the corresponding author upon reasonable request.
